# Long non-coding RNAs potentially function synergistically in the cellular reprogramming of SCNT embryos

**DOI:** 10.1186/s12864-018-5021-2

**Published:** 2018-08-23

**Authors:** Fengrui Wu, Yong Liu, Qingqing Wu, Dengkun Li, Ling Zhang, Xiaoqing Wu, Rong Wang, Di Zhang, Shaorong Gao, Wenyong Li

**Affiliations:** 1 Anhui Province Key Laboratory of Environmental Hormone and Reproduction, Anhui Province Key Laboratory of Embryo Development and Reproductive Regulation, Fuyang Normal University, Fuyang, China; 2 School of Life Sciences and Technology, Tongji University, Shanghai, China

**Keywords:** lncRNA, Single-cell RNA-seq, Somatic cell nuclear transfer, Cell reprogramming, Mouse embryo

## Abstract

**Background:**

Long non-coding RNAs (lncRNAs), a type of epigenetic regulator, are thought to play important roles in embryonic development in mice, and several developmental defects are associated with epigenetic modification disorders. The most dramatic epigenetic reprogramming event occurs during somatic cell nuclear transfer (SCNT) when the expression profile of a differentiated cell is abolished, and a newly embryo-specific expression profile is established. However, the molecular mechanism underlying somatic reprogramming remains unclear, and the dynamics and functions of lncRNAs in this process have not yet been illustrated, resulting in inefficient reprogramming.

**Results:**

In this study, 63 single-cell RNA-seq libraries were first generated and sequenced. A total of 7009 mouse polyadenylation lncRNAs (including 5204 novel lncRNAs) were obtained, and a comprehensive analysis of in vivo and SCNT mouse pre-implantation embryo lncRNAs was further performed based on our single-cell RNA sequencing data. Expression profile analysis revealed that lncRNAs were expressed in a developmental stage-specific manner during mouse early-stage embryonic development, whereas a more temporal and spatially specific expression pattern was identified in mouse SCNT embryos with changes in the state of chromatin during somatic cell reprogramming, leading to incomplete zygotic genome activation, oocyte to embryo transition and 2-cell to 4-cell transition. No obvious differences between other stages and mouse NTC or NTM embryos at the same stage were observed. Gene oncology (GO) enrichment analysis, Kyoto Encyclopedia of Genes and Genomes (KEGG) pathway analysis and weighted gene co-expression network analysis (WGCNA) of lncRNAs and their association with known protein-coding genes suggested that several lncRNAs and their associated with known protein-coding genes might be involved in mouse embryonic development and cell reprogramming.

**Conclusions:**

This is a novel report on the expression landscapes of lncRNAs of mouse NT embryos by scRNA-seq analysis. This study will provide insight into the molecular mechanism underlying the involvement of lncRNAs in mouse pre-implantation embryonic development and epigenetic reprogramming in mammalian species after SCNT-based cloning.

**Electronic supplementary material:**

The online version of this article (10.1186/s12864-018-5021-2) contains supplementary material, which is available to authorized users.

## Background

The loss of pluripotency gradually occurs from the zygote stage to organ maturation in multicellular eukaryotes. Under natural conditions, terminally differentiated cells are extremely stable and do not readily change into other cell types [1, 2]. Many studies have shown that some somatic cells can be reprogrammed by using somatic cell nuclear transfer (SCNT) [3–5], ectopic expression of a defined set of transcription factors (also named Yamanaka factors, OSKM factors) [6] and *piggyBac* transposons [7]. In general, cell fate is determined by the cell type-specific gene expression patterns during cell differentiation as well as nuclear reprogramming. For many years, many studies and reviews have focused on the control and maintenance of cellular identity during several developmental periods, especially focusing on gene transcriptional regulation and epigenetic modification [8–10]. Early SCNT experiments revealed the role of epigenetic regulation in determining reprogramming outcomes. The appearance of open chromatin is the key event of these processes [11, 12], followed by the important factors regulatory regions, facilitated chromatin remodelling and mediated gene expression [2]. While the use of multiple ectopic transcription factors in vitro has provided a more dynamic description of the regulators that coordinate the induction of silent genes, synergistic cooperation potentiates their ability to induce changes in cell fate [13]. Unfortunately, the reprogramming efficiency is still very low, especially for somatic cell reprogramming mediated by nuclear transplantation.

Long non-coding RNAs (lncRNAs) are a major type of non-coding RNA that closely resemble coding genes, as they have various exons and a polyadenylation tail but no open reading frame (ORF). Recently, an increasing number of studies have reported that lncRNAs exert critical functions at certain stages of cell differentiation and organism development, such as lung [14], liver [15], heart [16] and testis development [17]. To date, the expression patterns of lncRNAs in numerous cell lines and species of different classification orders have been systematically identified using high-throughput sequencing. In humans, lncRNAs involved in pre-implantation embryonic development (PED) as well as oocyte maturation, male germline development and zygotic genome activation (ZGA) were well-characterized based on public single-cell RNA sequencing (scRNA-seq) data [18, 19]. During three different stages of skeletal muscle development in chickens, lncRNAs and their target genes are potentially involved in cellular development, growth and proliferation, as determined using ingenuity pathway analysis [20]. In goats, lncRNAs have a strict tissue specificity and functional conservation during the early stage of skin development and pigmentation [21]. On the other hand, lncRNAs have been demonstrated to act as *cis* and *trans* elements with neighbouring or distal protein-coding genes and function as enhancers or alternative promoters for numerous genes in stem cell pluripotency and reprogramming [22]. For example, *Xist*, the most famous lncRNA, sufficiently triggers *cis* inactivation of the X chromosome during the 4-cell stage [23]. The lncRNA *Haunt* and its genomic locus were found to play discrete and opposing roles in regulating *HOXA* gene activation and embryonic stem cell (ESc) differentiation [24]. Epigenetic defects are well known to be the biggest barrier to SCNT-mediated cell reprogramming, and the promotion of reprogramming efficiency alters SCNT-associated epigenetic aberrations [25, 26]. Importantly, several studies have shown that lncRNAs can directly interact with chromatin-modifying enzymes and nucleosome-remodelling factors to alter the expression of specific genes [27–30]. Inspired by these reports, we speculate that lncRNAs may play important roles in the cell reprogramming process. However, the expression landscapes of lncRNAs in SCNT embryos and the regulation mechanism of lncRNAs in cell reprogramming remain unclear.

Herein, we first used our scRNA-seq data to estimate dynamic expression changes in lncRNAs found in fertilized embryos in vivo as well as in nucleus transfer (NT) embryos derived from cumulus cells (CCs) and mouse embryonic fibroblasts (MEFs) (named NTC and NTM, respectively) during mouse early development from the zygotic to the blastocyst stage. To further elucidate the functions of maternal and somatic cell-specific lncRNAs in development and direct cell reprogramming, mouse metaphase II (MII) oocytes and the two donor cell types were further sequenced and analysed by bioinformatic methods. Furthermore, several novel lncRNAs were also found in the present study. Our results not only provide a comprehensive analysis of the dynamic lncRNA landscapes in mouse embryos during pre-implantation development and cell reprogramming but also promote the application of NT technology in livestock production, therapeutic cloning and regenerative medicine.

## Construction and content

Somatic cell nuclear transfer (SCNT) is widely used in medicine, animal husbandry and other industries. However, certain limitations severely restrict the use of this technology, and the molecular mechanism of nuclear reprogramming remains unclear. Our initial idea for this study was to provide a foundation for studying the mechanism of nuclear reprogramming. To perform an exact syngeneic comparison of in vivo embryos and NT embryos, embryos were collected in vivo from female C57BL/6 mice that had been mated with male DBA/2 male. NTM embryos were generated using BDF1 MEF nuclei and MII oocyte cytoplasm. NTC embryos were generated with Cumulus cells collected from female BDF1 mice. The genetic background of all samples was BDF1. Then, 63 single-cell RNA-seq libraries were generated and sequenced. A total of 7009 mouse polyadenylated lncRNAs (including 5204 novel lncRNAs) were obtained. Differential expression analysis, GO and KEGG pathway enrichment analyses and weighted co-expression network analysis were conducted to explore the potential roles of lncRNAs in cell reprogramming.

## Utility

Till now, there are few reports on the expression landscapes of lncRNAs of mouse NT embryos. In this study, we provided the expression profiles of 7009 mouse polyadenylation lncRNAs (including 5204 novel lncRNAs) obtained from in vivo and SCNT mouse pre-implantation embryo. Users can download the rawdata of these samples based on single-cell RNA sequencing from NCBI to further explore the mechanism of mouse embryonic development, especially the potential roles of lncRNAs in nuclear reprogramming.

## Results

### Overview of scRNA-seq

**Fig. 1:**
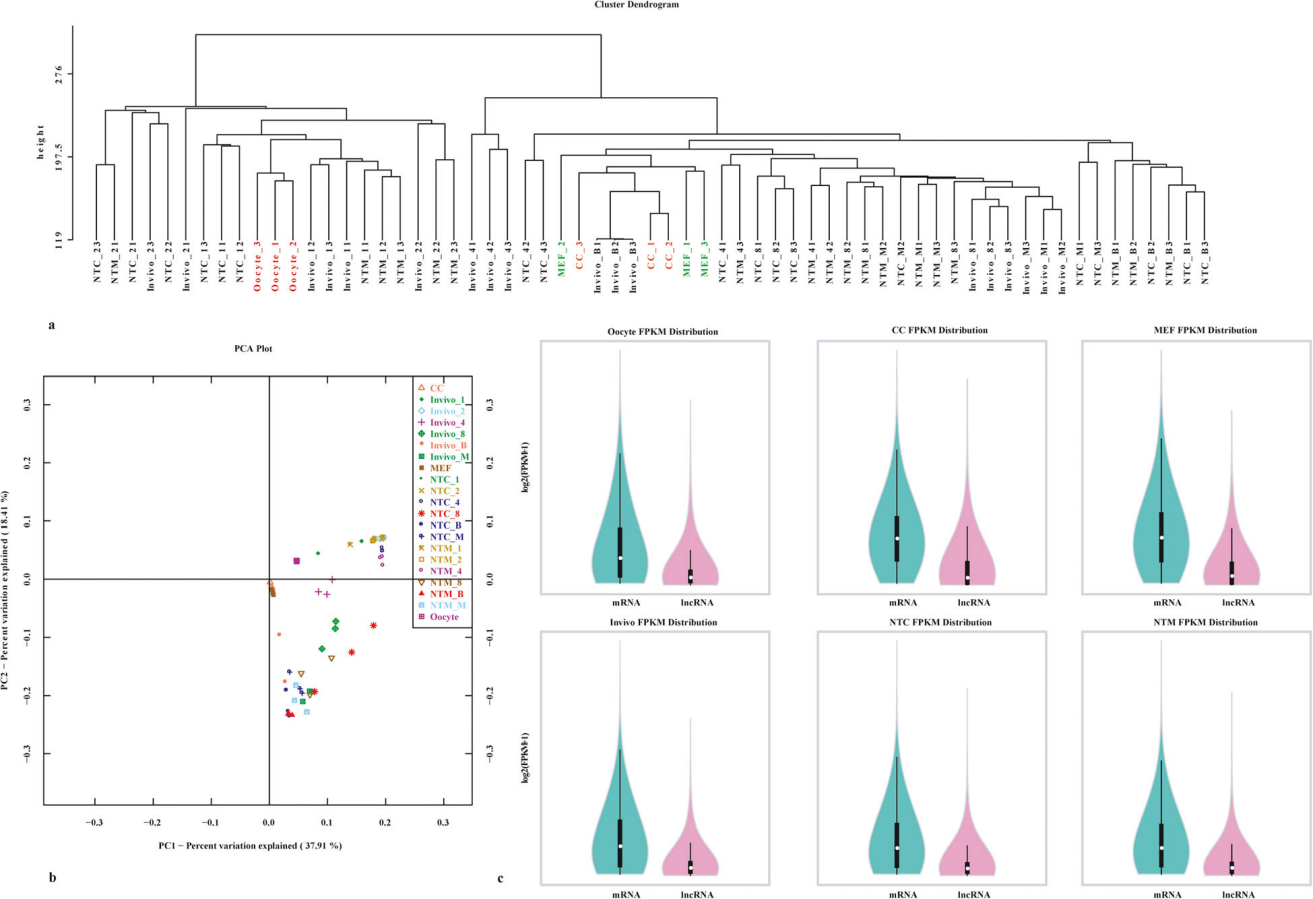
Overview of lncRNAs in MII oocyte, CC, MEF, in vivo and two nuclear donor cell NT pre-implantation mouse embryos at different stages by single-cell RNA sequencing. **a**, Unsupervised hierarchical clustering across MII oocyte, CC, MEF and all types of pre-implantation embryos. **b**, Principal component analysis of the lncRNA expression pattern of each sample. Zygotes and 2-cell in vivo embryos were clustered together with those of the NTC and NTM groups. The numbers of samples for each stage are also indicated in the PCA. C, FPKM distributions between mRNAs with lncRNAs in MII oocyte, CC, MEF, in vivo, NTC and NTM embryos

To identify lncRNA expression profiles, we constructed 63 cDNA libraries that were derived from one MII oocyte, cumulus cell, and MEF in vivo and two nuclear donor cell mouse NT embryos at different stages prior to implantation. Three biological replicates of each sample were used. The libraries were sequenced using an Illumina HiSeq 2500 platform, and 150-bp paired-end reads were generated. The mapped sequences in each library were assembled, and after discarding adaptor sequences, low-quality reads, and short and unreliable transcripts, a total of 7008 polyadenylated mouse lncRNAs were obtained. To our knowledge, this is a comprehensive and in-depth scRNA-seq analysis of lncRNAs in mouse NT embryos. Unsupervised hierarchal clustering analysis was conducted to elucidate the distinctions between the different samples and their biological relevance. Six groups, including oocytes, CCs, MEFs, in vivo embryos, NTC embryos and NTM embryos, were divided according to the lncRNA expression profiles of all samples (Fig. 1a). Principal component analysis (PCA) further clustered the oocytes, CCs, MEFs, in vivo embryos and NT embryos by developmental stage and embryonic type, effectively reconstructing the dynamics of pre-implantation development (Fig. 1b) and indicating that the expression profiles of the lncRNAs in zygotes and 2-cell embryos were similar between the in vivo group and the NT group (Fig. 1a and b). Intriguingly, cluster analysis showed that the zygotes (1-cell stage) and 2-cell embryos of the in vivo and NT groups clustered closely together with oocytes, suggesting that maternal lncRNAs might play important roles in all groups prior to the 2-cell stage. Comparisons between mature oocytes and 1-cell in vivo embryos identified 70 differentially expressed lncRNAs, 49 and 21 of which were up- and down-regulated, respectively, in 1-cell embryos (Additional files 1 and 2). Notably, the lncRNAs *Pvt1* and *Kantr* (non-coding transcript adjacent to Kdm5c) were identified as up- and down-regulated (q < 0.001, |log2 fold change| > 2), respectively, indicating that the TGF-β/Smad pathway and histone methylation may play important roles during the oocyte-to-embryo transition (OET). As shown in Fig. 1c, a comparison of the FPKM distributions between mRNAs and lncRNAs showed that the expression levels of lncRNAs were lower than those of mRNAs in each sample, indicating that lncRNAs may exert critical functions as *cis-* or *trans-*acting elements by binding to and regulating the expression of mRNAs during specific biological events.

### Dramatic temporal and spatial expression patterns of lncRNAs in pre-implantation in vivo and NT mouse embryos

**Fig. 2:**
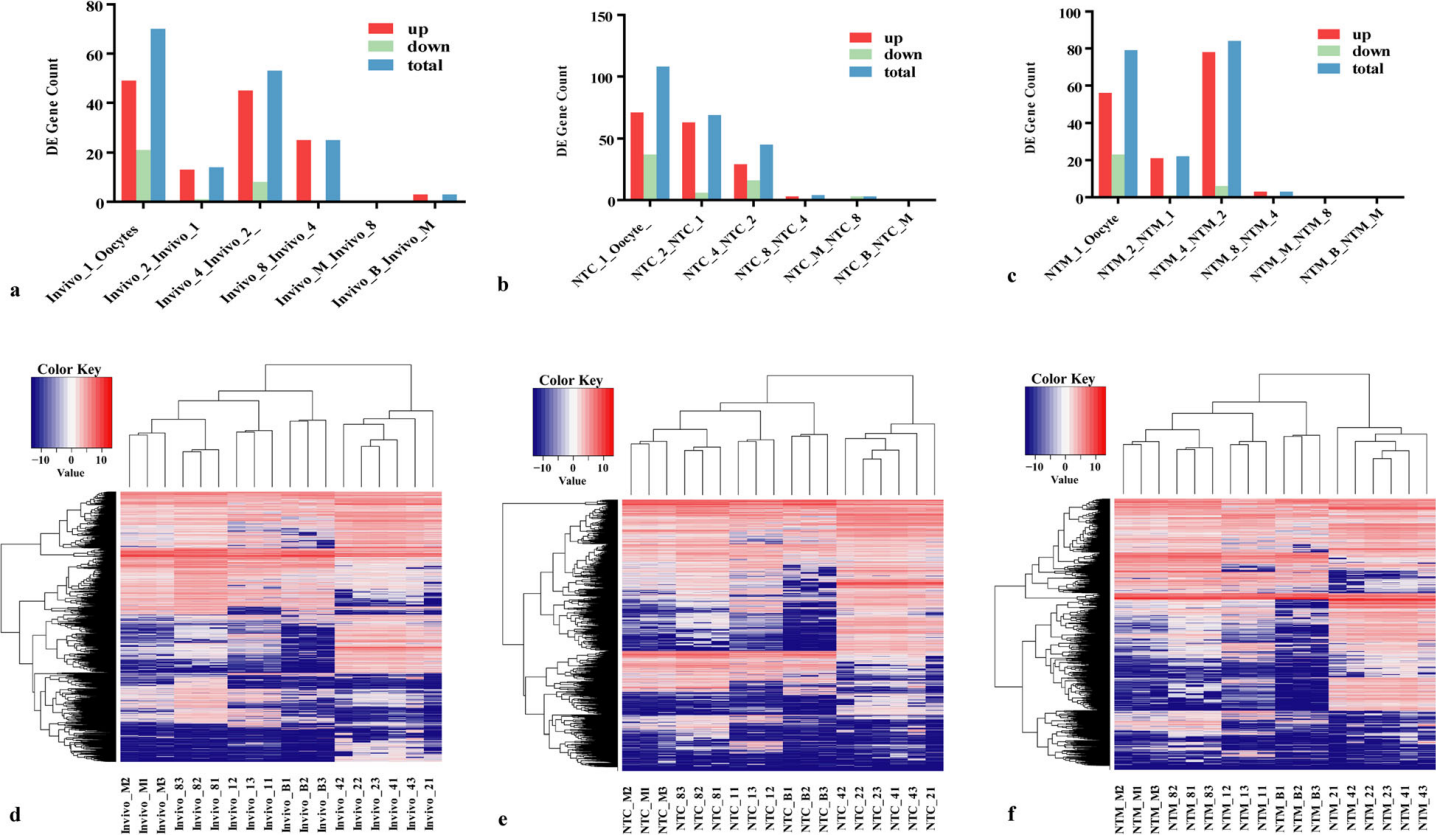
Dynamic lncRNA expression landscape of in vivo embryos and two types of SCNT mouse pre-implantation embryos. **a**, **b** and **c**, Differentially expressed lncRNA gene counts in in vivo embryos and two types of SCNT mouse pre-implantation embryos. **d**, **e** and **f**, Heatmap showing the temporal and spatial profiles of differentially expressed lncRNAs in in vivo embryos and two types of SCNT mouse pre-implantation embryos. Blue indicates lower expression, red indicates higher expression

To explore the temporal expression patterns of pre-implantation in vivo and mouse NT embryos, RNA sequencing data from all 63 samples were analysed via DEGseq and DESeq. First, we conducted vertical differential expression analysis across the in vivo, NTC and NTM groups. Dramatic changes in lncRNA expression were observed, peaking at the 2-cell to 4-cell stage in the in vivo samples (45 and 88 of which were up- and down-regulated, respectively, Fig. 2a and d, Additional file 3) (|log2 fold change| > 2, *p*-value< 0.001); these lncRNAs included the cleavage-specific lncRNAs *Smkr-ps*, *Mkln1os*, *Redrum*, *Platr27*, *Platr23*, *Snhg12*, *Malat1* and *Fbxw27*, indicating that most maternal lncRNAs were degraded after ZGA. However, the large-scale down-regulation of gene expression was not found in NT embryos. In the NTM groups, 78 and 6 lncRNAs were up- and down-regulated, respectively (Fig. 2c and f, Additional file 4), and included *Neat1*, *Platr17*, *Tunar* and *Snhg20*. By contrast, the differences in the NTC group peaked at the zygote to 2-cell stage (63 and 6 of which were up- and down-regulated, respectively, Fig. 2b and e, Additional file 5), and the lncRNAs included *Drr1*, *Lncenc1* and *Vmn2r-ps111*. These findings suggest that this embryo-specific lncRNA expression pattern in NT embryos cannot be gradually established due to incomplete ZGA. As shown in Fig. 2d, e and f, some lncRNAs were expressed at high levels at the zygote and 2-cell stage in the in vivo, NTC and NTM groups but were expressed at low levels at the other stages, indicating that these lncRNAs might be gradually degraded and repressed after ZGA. Notably, some lncRNAs that were expressed at low levels in the in vivo group at the zygote and 2-cell stage progressively increased from the 4-cell stage onward; these lncRNAs included *Fam169b* and *Epb41l4aos*. These findings suggested that these lncRNAs might be related to cell-fate decisions during mouse pre-implantation embryonic development, due to this specific gene expression pattern in response to the period of cell differentiation. However, a similar phenomenon did not appear in the NTC or NTM groups (Additional file 6).

**Fig. 3:**
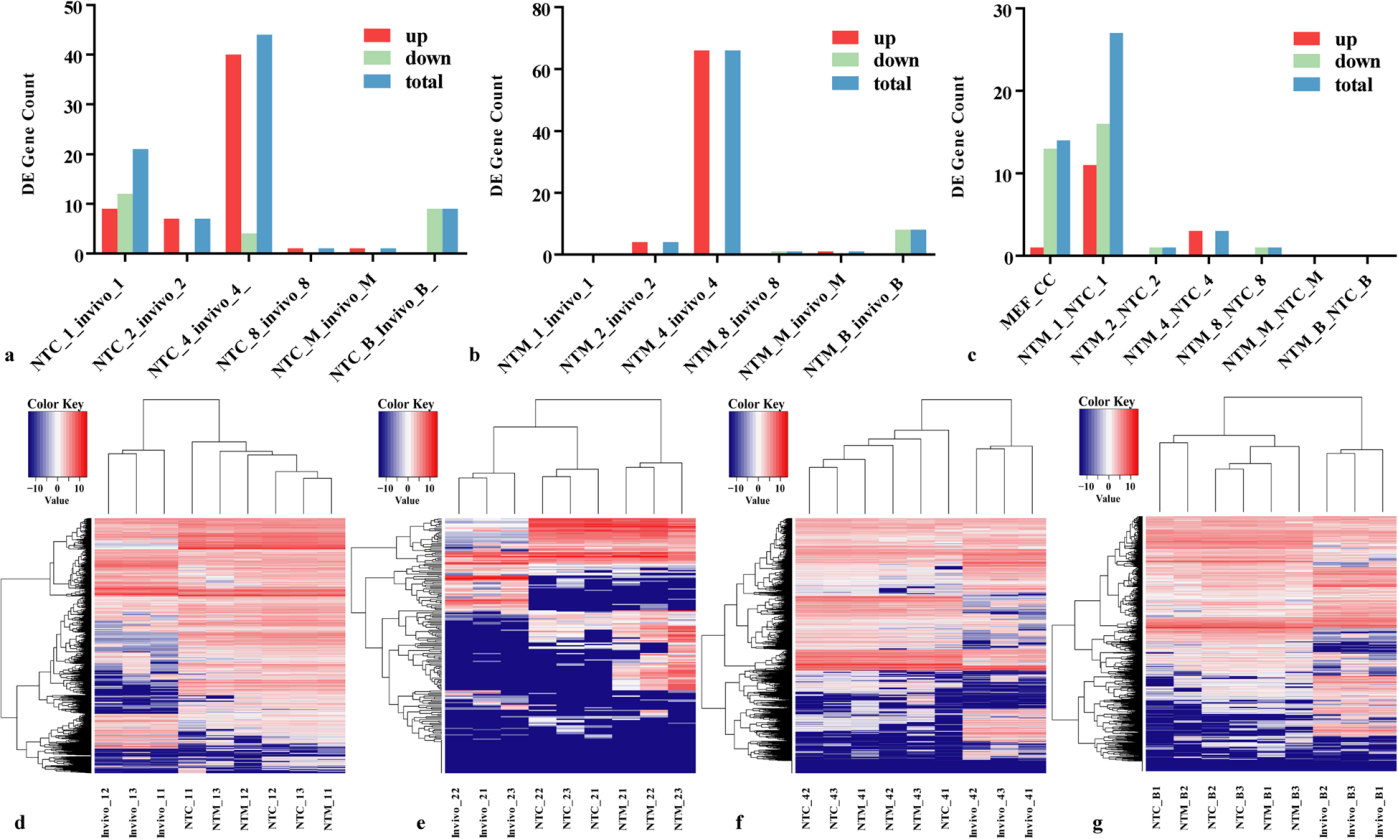
Cross-comparison of in vivo embryos and two types of SCNT embryos at six stages of mouse pre-implantation embryo development. **a**, **b** and **c**, Differentially expressed lncRNA gene counts in in vivo embryos and two types of SCNT mouse pre-implantation embryos. **d**, **e**, **f**, and **g**, Heatmap showing differentially expressed lncRNA expression profiles in in vivo embryos and two types of SCNT mouse embryos at the zygotic, 2-cell, 4-cell and blastocyst stages

Next, horizontal comparisons were performed to identify the differential expression patterns across the in vivo, NTC and NTM groups (Fig. 3a, and b). The period with the largest number of differentially expressed lncRNAs between the NTC/NTM and in vivo embryos was the 4-cell stage. In the NTC vs in vivo group, 40 up-regulated lncRNAs and 4 down-regulated lncRNAs (|log2 fold change| > 2, *P*-value< 0.001) were identified at the 4-cell stage (Additional file 7); these lncRNAs included *Crnde*, *Snhg18*, *BC051077*, *Gm16702*, *Gm28875* and *Gm26905*. In the NTM vs in vivo group, only 66 up-regulated lncRNAs and no down-regulated lncRNAs were found; the identified lncRNAs included *c78283*, *Dubr*, *Lppos*, *Gm43672*, *Gm26760* and *Gm12249* (Additional file 8). However, a comparison of the NTM group with the NTC group showed that the number of differentially expressed lncRNAs at the zygote stage was greater than that at other stages (Fig. 3c), indicating that different donor-cell-specific lncRNA expression patterns directly affect the capacity for late embryonic development. Furthermore, unsupervised hierarchal clustering and heat map analyses showed that in NT embryos, the expression of lncRNAs from the 4-cell stage onwards exhibited expression patterns that were similar to embryo-specific expression patterns at each time point (Fig. 3d, e, f and g), indicating that the efficiency of somatic cell reprogramming might be directly affected by these differentially expressed lncRNAs.

**Fig. 4:**
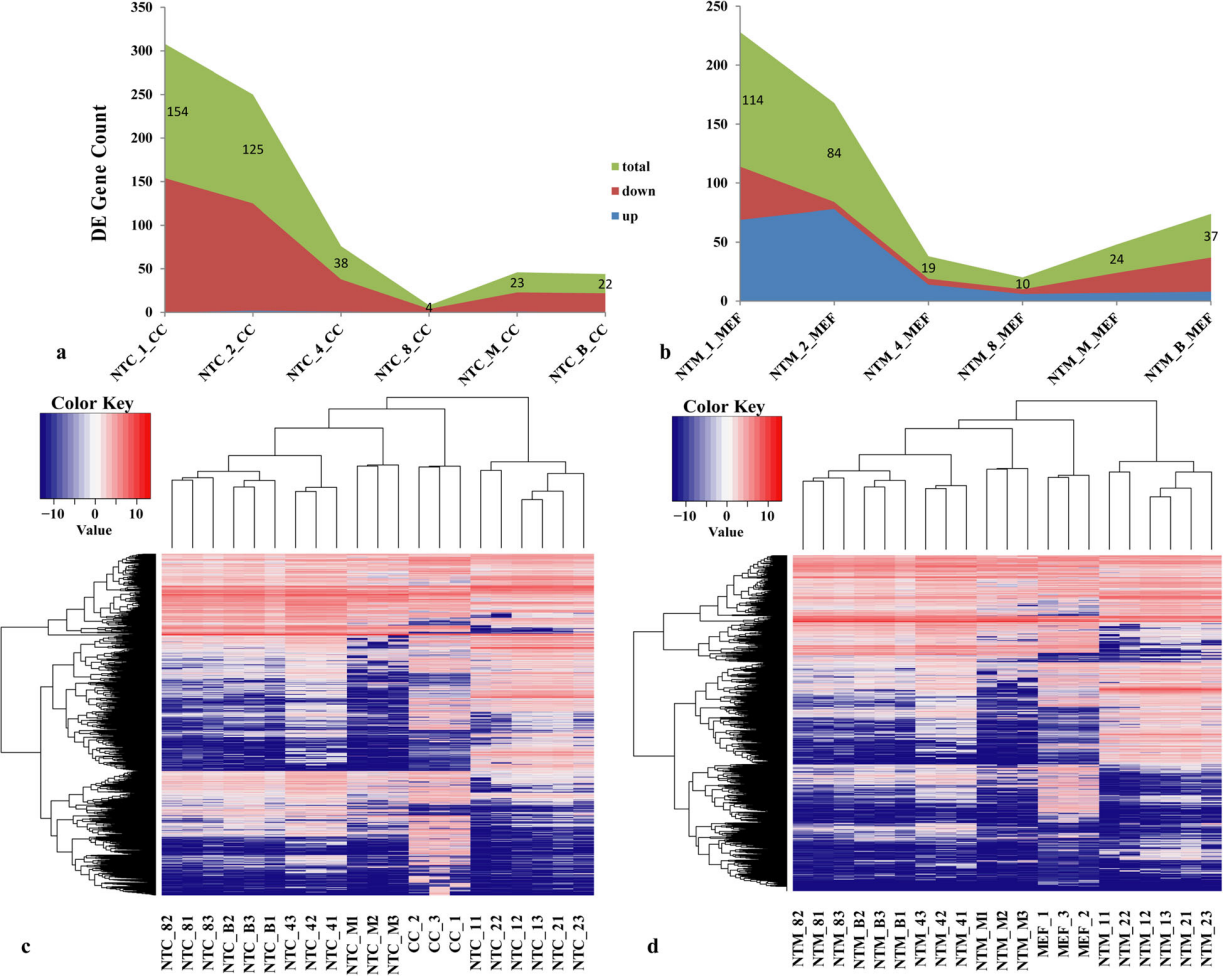
Comparison of NT embryos and their own donor cells at different developmental stages of mouse pre-implantation embryonic development. **a** and **b**, Dynamic changes in differentially expressed lncRNAs in NTC and NTM embryos and their own donor cells at six developmental stages of mouse pre-implantation embryonic development. **c** and **d**, Heatmap showing differentially expressed lncRNA expression profiles in NTC and NTM embryos and their own donor cells at six developmental stages of mouse pre-implantation embryonic development

The lncRNAs that were differentially expressed between the CC and NTC groups as well as between the MEF and NTM groups were further characterized via bioinformatic analyses (Fig. 4). The total number of differentially expressed lncRNAs in pre-implantation NT embryos peaked at the zygotic stage and decreased at the 8-cell stage; these lncRNAs included *Neat1*, *Ftx*, *Meg3*, *Jpx*, *lockd*, *Mir17hg*, *H19*, *Carmn*, *Mir155hg* and *Lncenc1*. These findings suggested that somatic-cell-specific lncRNAs progressively decrease and are reprogrammed after ZGA (Fig. 4a and b). Moreover, the trends in both the CC vs NTC and the MEF vs NTM embryo comparisons were consistent with each other (Fig. 4c and d), indicating that the establishment of embryo-specific lncRNA expression patterns might be independent of donor cell type.

### Functions of lncRNAs in cell reprogramming

**Fig. 5:**
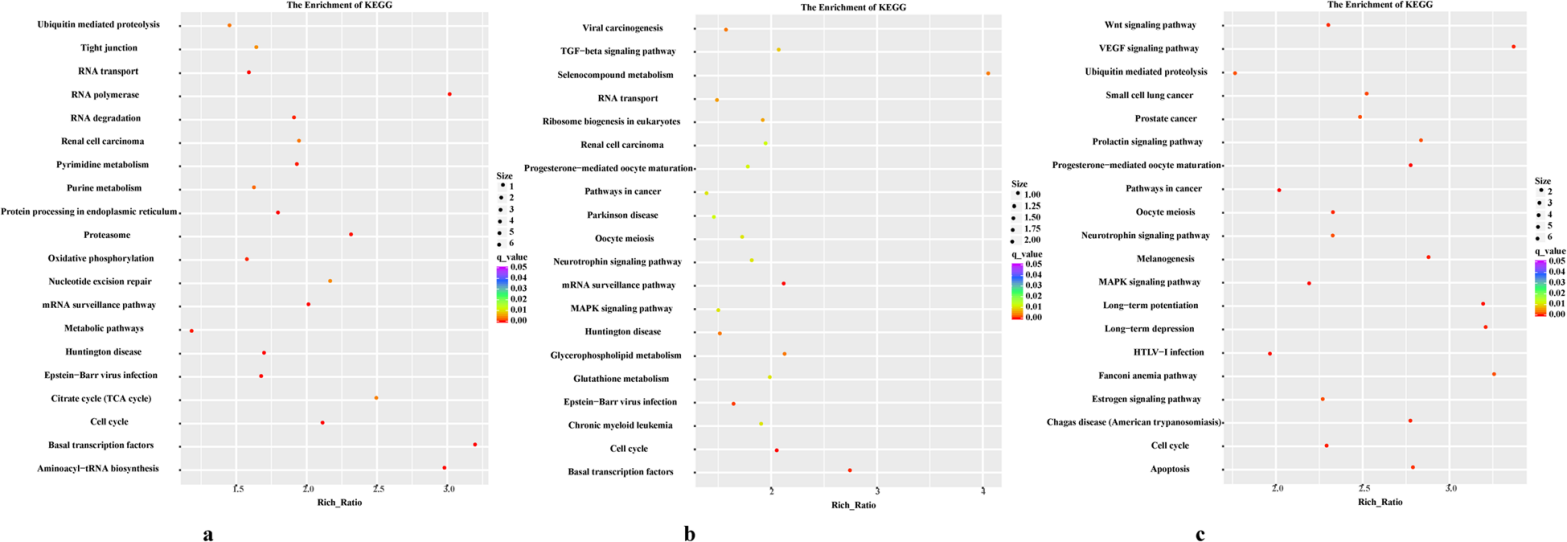
KEGG pathway analysis of lncRNAs with the top 20 enrichment scores. **a**, **b** and **c**, KEGG enrichments of lncRNAs and their associations with known protein-coding genes in the mouse in vivo embryo and two types of NT embryos during pre-implantation development and cell reprogramming, respectively

To investigate the potential roles of lncRNAs in the in vivo, NTC and NTM pre-implantation mouse embryos, KEGG pathway analysis was conducted. A comparison of the in vivo and NTC groups suggested that significantly enriched protein-coding neighbours are involved in RNA transport, RNA degradation and basal transcription (Fig. 5a), while the mRNA surveillance and cell cycle pathways were highlighted in the comparison between the in vivo and NTM groups (Fig. 5b), indicating that the slight cell cycle mismatch between G0/G1-arrested donor nuclei and MII oocytes might be not completely corrected after SCNT, leading to the formation of a closed chromatin state. Interestingly, in the comparison of the NTC and NTM groups, all terms, especially the VEGF signalling pathway, progesterone-mediated oocyte maturation and oocyte meiosis, exhibited significant differences and higher ratios (Fig. 5c).

**Fig. 6:**
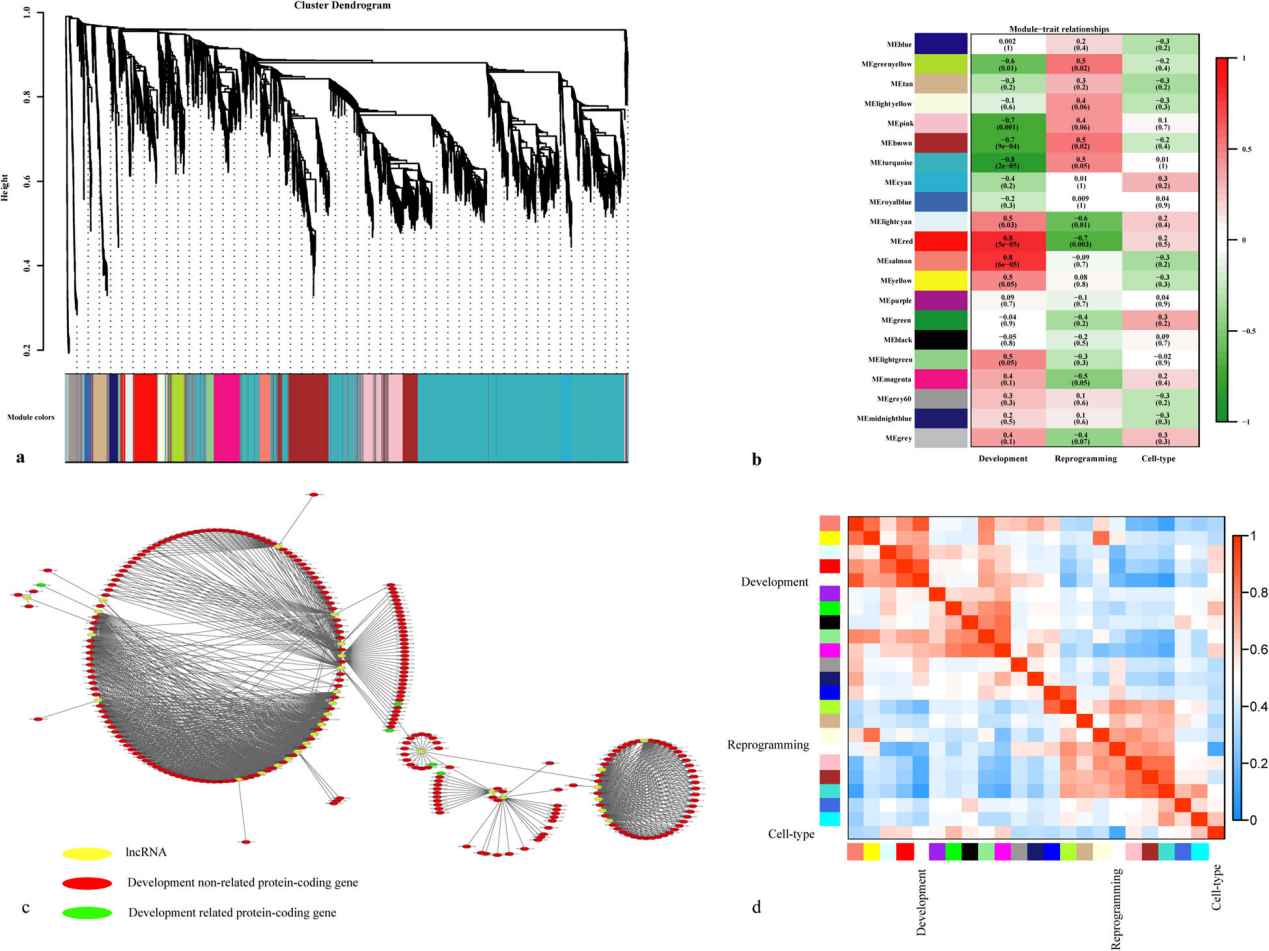
Network analysis of lncRNAs and their associations with known protein-coding genes in the mouse in vivo embryo and NT embryos during pre-implantation development and cell reprogramming. **a**, Hierarchical cluster tree showing co-expression modules identified using WGCNA. The modules correspond to branches and are labelled by colours, as indicated by the colour band underneath the tree. **b**, Heatmap showing correlations followed by the *P*-values (*p* < 0.05) in parentheses between modules, development, reprogramming and donor cell type. The colour of each square corresponds to the degree of correlation: positive correlation, red; negative correlation, green; no correlation, white. **c**, Co-expressed lncRNAs and protein-coding genes in the largest module that are closely related to mouse embryonic development. **d**, Correlation plot of 20 module genes with development, reprogramming and donor cell type. Each row and column in the heatmap corresponds to 1 module gene (labeled by the same colour as that in a). In the heatmap, the red colour represents high adjacency (positive correlation), and the blue colour represents low adjacency (negative correlation)

WGCNA is a powerful ‘guilt-by-association’-based method to predict the functions of and associations between lncRNAs and mRNAs (Fig. 6a). By clustering correlated genes together, 21 co-expressed gene modules were identified from the in vivo and NT pre-implantation mouse embryos. Ten of twenty-one modules were correlated (correlation> 0.4) with specific developmental stages, reprogramming or donor cell types (Fig. 6a and b). Notably, 2 of the 10 modules (red and salmon modules, correlation = 0.8, *p*-value< 10^− 4^) were highly correlated with development. Five protein-coding genes involved in cell reprogramming, including SH3-domain binding protein 5 (*Sh3bp5*), spinocerebellar ataxia type 10 (*Ataxin-10*), procollagen-lysine, 2-oxoglutarate 5-dioxygenase 2) (*Plod2*), carboxypeptidase E (*Cpe*) and serine (or cysteine) peptidase inhibitor, clade B, member 6b (*Serpinb6b*), were identified via whole-network analysis (Fig. 6c). In all the in vivo and pre-implantation mouse NT embryos, both the red and salmon modules contained many genes and were highly associated with developmental processes (Fig. 6d). An analysis of GO term enrichment within these two modules was performed (Additional files 9 and 10). Genes in the red module (Additional file 11) were enriched for RNA polymerase II transcription, the positive regulation of histone modification and protein deacylation, while genes in the salmon module were enriched for cellular response to DNA damage stimuli and the post-transcriptional regulation of gene expression (Additional file 12). These results suggest that the lncRNAs in these two modules might be involved in epigenetic modification during pre-implantation embryo development (PED) in mice, especially in forming an open chromatin state to improve gene transcription.

### Novel lncRNAs were identified in all samples

**Fig. 7:**
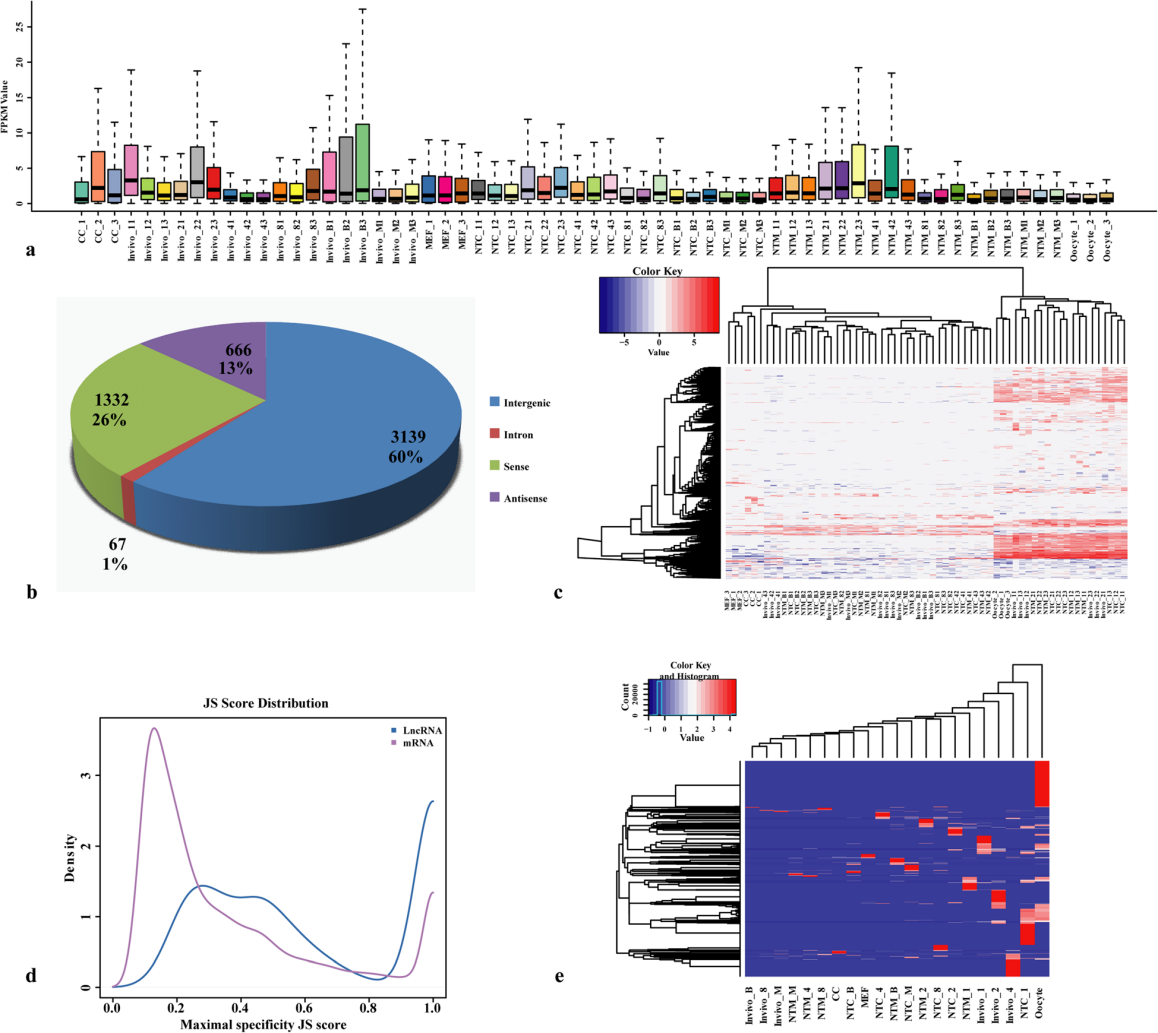
Identification of novel lncRNAs in the mouse oocyte, cumulus cell, MEF in vivo and two types of NT embryos during pre-implantation development. **a**, Boxplot indicating the distribution of novel lncRNA FPKMs in all 63 samples. **b**, pie chart showing the numbers and percentages of different types in these samples. **c**, Bi-clustering of the log-normalized FPKM values estimated by Cufflinks for ENSEMBL lincRNA genes across the listed pre-implantation developmental stages. **d**, Distributions of maximal tissue specificity scores calculated for the lncRNAs and protein-coding transcripts in all samples. **e**, Heat map of novel differentially expressed lncRNAs in all 63 samples (JS score > 0.5)

All 63 samples were searched for novel lncRNAs not found in the ENSEMBL (http://asia.ensembl.org/index.html) or Noncode v 3.0 (http://www.noncode.org/index.php) lncRNA databases. As shown in Fig. 7a, to display the trends of the distribution of these novel lncRNAs, a box plot was drawn based on the expression levels of novel lncRNAs in each sample. These novel lncRNAs were not expressed and had different FPKMs in each sample. For example, we found 712 novel lncRNAs that were expressed at FPKM values> 1 and 97 that were expressed at FPKM values> 0.5. The average FPKM value of the novel lncRNAs in the in vivo B group was higher than those in the other groups, indicating that these novel lncRNAs might play important roles in later developmental stages. After discarding transcripts that were less than 200 bp in length or had one exon, using three-read coverage, we used coding potential calculator (CPC) and coding-non-coding index (CNCI) software to evaluate the coding potentials of the remaining transcripts with three-read coverage; we identified 5204 novel lncRNAs (Fig. 7b), including 3139 novel intergenic, 67 novel intronic, 1332 novel sense and 666 novel antisense lncRNAs. To address the different expression profiles of these novel lncRNAs across the samples (Fig. 7c), unsupervised hierarchal clustering analysis was conducted using R software (v 3.0); the results were consistent with those of known lncRNAs. The tissue-specific patterns of these novel lncRNAs were further evaluated based on Jensen-Shannon divergence as previously described. Heat maps and density plots of tissue specificity scores showed significantly higher tissue specificities for the newly identified lncRNAs than for protein-coding genes (Fig. 7d, e and f). To explore the potential functions of these novel lncRNAs, their *cis* or *trans* targets were predicted. The results showed that many novel lncRNAs could regulate their neighbouring coding genes in *trans* during cell reprogramming (Pearson correlation coefficient < 0.9), while only 11, such as *Ptchd3*, *Kcnv2* and *Wdr74*, acted in *cis*. GO enrichment analysis showed that *trans* target genes of the novel lncRNAs were enriched for extracellular structure organization, response to growth factor and steroid metabolic process (Additional file 13). Therefore, we believe that these novel lncRNAs are correlated with developmental-stage-specific regulation.

## Discussion

Many studies have reported that lncRNAs can play important roles in various biological processes, such as gene transcription, translation, and metabolism. The possible functions of lncRNAs in early embryonic development and cell reprogramming have gradually become research hotspots for scientists [31, 32]. Profiling the lncRNA transcriptomes of some microscale biological samples is difficult because of technical obstacles, such as the analysis of lncRNAs expressed at lower levels and in small cellular amounts [16, 18], as well as those in NT embryos.

Recent studies have reported that the parental chromosomes quickly open shortly after fertilization and reach a highly open state at the early zygote stage, followed by decreased openness after the late zygote stage. Then, the degree of opening gradually increases again after the 2-cell stage and reaches its highest point at the blastocyst stage during the development of pre-implantation embryos [33–35]. However, chromatin state can be controlled by lncRNAs, and lncRNA expression can also be controlled by chromatin-remodelling factors. Nuclear-transfer-mediated reprogramming is the most efficient reprogramming method. Assembling the ideal chromatin state has always been an important factor restricting the efficiency of nuclear-transfer-mediated nuclear reprogramming. Therefore, in the present study, we used our scRNA-seq data to systematically analyse the dynamic landscape of lncRNA expression in in vivo embryos and two types of SCNT mouse pre-implantation embryos. The lncRNAs expressed in these embryos exhibited obvious developmental stage dependence that corresponded to changes in chromatin configuration [33] (Guo et al., 2017). Based on PCA and an unsupervised hierarchical clustering analysis of the lncRNA expression profiles, oocytes and three types of embryos at the 1-cell stage closely clustered together, indicating that maternal lncRNAs might be essential factors at the 1-cell stage when the transcription of the entire embryos is quiescent. Others have previously shown that the lncRNA *Pvt1* could bind the histone-lysine N-methyltransferase EZH2 to change histone modification patterns [36]. The lncRNAs *Pvt1* and *Kantr* were identified as important DE genes in the in vivo group, but not in NT embryos, during the OET. These maternal factors in oocytes reportedly have critical roles in nuclear reprogramming and early embryonic development [37, 38]. Our results further support this classic point of view and are also consistent with the expression patterns of mRNAs in early human and mouse embryos [39]. However, the total number of differentially expressed lncRNAs in the zygote and oocyte comparison was much larger than that in the NTC and oocyte comparison, suggesting maternal lncRNAs cannot be gradually degraded, and the transformation of a differentiated oocyte into a developing embryo was subsequently blocked. The transcription of lncRNAs might be suppressed by SCNT, leading to the abnormal initiation of ZGA. Interestingly, the lncRNAs in the 1-cell, 8-cell, morula and blastocyst groups from all three embryo types clustered together and were separated from those of the 2-cell and 4-cell embryos. In mouse embryos at the cleavage stage, lncRNAs are expressed in a developmental-stage-specific manner [23, 40, 41]. This phenomenon was also found in our study, indicating that the developmental and cell-reprogramming processes are regulated by stage-specific lncRNAs. This result is consistent with the mRNA and protein expression patterns in humans and mice [39, 42].

Next, the differences in lncRNA expression in these embryos were analysed by making vertical comparisons, which revealed that the maximum number of differentially expressed lncRNAs peaked at the 4-cell stage. In mice, ZGA initiates during the late 1-cell stage (minor ZGA), followed by major gene activation (major ZGA) at the 2-cell stage. Many up-regulated lncRNAs were found in the comparison between the NT and in vivo embryos, while few were found in the comparison of the NTM and NTC groups. These observations indicate that embryo-specific lncRNAs are not reprogrammed and are expressed in NT embryos and that somatic memory in cloned embryos is not erased after major ZGA. For example, the lncRNA *Crnde* can regulate PI3K/Akt/β-catenin and Notch1 signalling to maintain the pluripotency of stem cells and regulate cell reprogramming [43, 44]. The results were consistent with the reported dramatic changes in expression during the first cleavage division of the zygote [40, 45] and the OET [31]. Therefore, we believe that these lncRNAs may regulate cell reprogramming by regulating nucleosome and chromatin assembly, especially from the 2-cell stage onward. Moreover, comparing the two donor cells with NTC and NTM embryos further supported this idea. Although the rate of blastocyst development in the NT embryos was substantially improved by the injection of H3K9me3 demethylase and treatment with the histone deacetylase inhibitor trichostatin A at the one-cell stage [26, 46] in mouse and non-human primate, the implantation efficiency was still low. Many down-regulated lncRNAs were found when comparing the in vivo and NT embryos at the blastocyst stage, whereas no differentially expressed lncRNAs were found when comparing the NTC and NTM groups, suggesting that lncRNAs are also important for the implantation and development of transplanted SCNT embryos.

To obtain a comprehensive understanding of lncRNAs and their association with known protein-coding genes and to predict the functions of lncRNAs in embryonic development and cell reprogramming, GO and KEGG analyses were conducted. GO and KEGG analyses of the lncRNAs in different embryo types showed that the differentially expressed lncRNAs tended to function in a variety of processes, including RNA transport, degradation, oocyte meiosis, the cell cycle, and the TGF-β and Wnt signalling pathways, and they provide energy and materials to support the basic requirements for embryonic growth and cell reprograming. Previous studies have reported that culture conditions and environmental embryonic changes could induce histone modifications [47–50]. Progesterone-mediated oocyte maturation and the oestrogen signalling pathway were significantly enriched only in NT embryos, indicating that the epigenetic reprogramming of lncRNAs in NT embryos might be abnormally disturbed by environmental stress. Two particularly obvious modules (q < 10^− 4^) with coding genes with known functions were identified by WGCNA, and GO enrichment analysis of the lncRNAs in these modules further revealed distinct biological functions. These results not only help identify important lncRNAs during early embryonic development and cell reprogramming but also clarify the essential roles of epigenetic modifications.

## Conclusions

Taken together, although many studies have been reported the dynamic mRNA and protein expression landscapes in mouse pre-implantation embryos, little is known about ncRNAs, especially regarding the lncRNAs expression landscapes in cloned embryos. In this work, the polyadenylation lncRNAs expression landscapes in mouse in vivo and NT embryos during six stages of pre-implantation development were illustrated by the single-cell RNA sequencing. Using horizontal and vertical analyses, we initially revealed defects in lncRNA-guided epigenetic reprogramming in cloned embryos. Moreover, several lncRNAs and their associated with known protein-coding genes were identified by WGCNA, highlighting the functions of lncRNAs to further our understanding of pre-implantation development and cell reprogramming. Our data not only provide a basic dataset for further mechanistic studies on lncRNAs in pre-implantation embryos but also suggest more factors that are involved in early embryonic development and cell reprogramming at levels other than transcription, translation and post-translation.

## Methods

### Ethics statement

The study protocol was approved by the Animal Care and Management Committee of Fuyang Normal University. All animal manipulations were performed according to the guidelines of the Animal Care Committee.

### Embryo collection and nuclear transfer

Embryo collection and nuclear transfer were performed according to methods described elsewhere [51].

### Single-cell RNA-seq library generation

The scRNA-seq method followed protocols established in previously published studies [52–54]. Briefly, the harvested single MEF, CC, MII oocyte, and pre-implantation embryos of the in vivo, NTM and NTC groups were washed several times in 0.5% BSA-PBS (Sigma) solution and subsequently selected and transferred into lysate buffer by a mouth pipette. A diluted ERCC mix (Life Technologies 4,456,740) was spiked into the lysis buffer of each sample, and reverse transcription was performed directly on the cytoplasmic lysate. Terminal deoxynucleotidyl transferase was then used to add a poly (A) tail to the 3′ end of the first-strand cDNA. The total cDNA library of the single cell was then amplified in 18–20 cycles for the library construction, and the amplified cDNA was fragmented using the Covaris sonicator (Covaris S220, Woburn, MA, USA). To generate the sequencing libraries, the KAPA Hyper DNA Library Prep Kit (KK8504, Kapa Biosystems) was used following the manufacturer’s instructions. All adapters are listed in Additional file 14. Paired-end 150-bp sequencing was further performed on the HiSeq 2500 platform (Illumina) at Annoroad Gene Technology Corporation., Ltd. (Beijing, China).

### Mapping the sequencing reads to reference genomes

The reference mouse (mm9) genomes and annotation file were downloaded from the ENSEMBL database (http://www.ensembl.org/index.html). In addition, clean data were mapped to the reference genome using HISAT2 (http://ccb.jhu.edu/software/hisat2/index.shtml) with default parameters, which can identify exon-exon junctions by splitting and re-mapping the mapped reads to the reference genome. Aligned reads from HISAT2 were then separately assembled into transcripts by Cufflinks (version V2.2.1) and then evaluated using RseQC (v 2.3.4) to remove low-quality samples, samples with mapping efficiencies less than 30%, and mapped read counts less than 2 M, and intron read percentages greater than 10% were discarded from downstream analyses.

### Quantitation of gene expression

Read counts for each gene in each sample were counted by HTSeq, and fragments per kilobase million mapped reads (FPKM) were then calculated to represent the expression level of genes in each sample using the formula shown below:

$$ FPKM=\frac{10^{6\ast }F}{NL/{10}^3} $$

where F is the number of fragments in a sample assigned to a certain gene, N is the total number of mapped reads in the sample, and L is the length of the gene. Thus, FPKMs are adjusted for coverage and gene length and can be directly compared.

### Differential expression analysis

DEGseq (http://www.bioconductor.org/packages/release/bioc/html/DEGseq.html) or DESeq (http://www.bioconductor.org/packages/release/bioc/html/DESeq.html) was used for the differential expression analysis of two samples with replicates. Assuming that the number of reads from a gene (or transcript isoform) follows a binomial distribution, DEGseq is proposed based on the MA-plot and widely used for differential expression analysis. A *P*-value was assigned to each gene and adjusted by the Benjamini and Hochberg (BH) method. Genes with q ≤ 0.05 and |log2_ratio| ≥ 1 were identified as being differentially expressed.

### GO and KEGG pathway enrichment analyses

To investigate genes from one gene ontology GO term (http://geneontology.org/), a hypergeometric *p*-value was calculated and adjusted as a q-value, where the background was set to be genes in the whole genome. GO terms with *q* < 0.05 were considered significantly enriched, and GO enrichment analysis elucidated the biological functions of the differentially expressed genes (DEGs). The log10 value (*p*-value) denotes enrichment scores that represent the significance of GO term enrichment among DEGs. Kyoto Encyclopedia of Genes and Genomes (KEGG, http://www.kegg.jp/) pathway analysis was also performed to predict the molecular interactions and reaction networks associated with differentially regulated genes. Using the same method as that used for GO enrichment analysis, significantly enriched KEGG pathways were identified.

### Weighted co-expression network

A weighted gene co-expression network was constructed with differentially expressed mRNAs and lncRNAs using the weighted correlation network analysis (WGCNA) package [55] in R. Briefly, genome-wide gene expression data were initially filtrated by measuring the consistency of gene expression profiles by the Pearson correlation in the data processing step. The power function adjacent to the Pearson correlation matrix was then utilized to transform the data into weighted gene co-expression networks, where a network module represented a cluster of closely interconnected genes. Finally, the adjacency matrix, a measurement of topology similarity, was converted into the topological overlap matrix (TOM), and modules were detected by clustering analysis.

## Additional files

Additional file 1: DE lncRNAs with significant difference compared zygote with oocytes. (XLSX 17 kb)

Additional file 2: Heat map (a) and Volcano map (b) show the DE lncRNAs levels when compared with oocyte and zygote. Red represents up-regulated significantly, while green represents down-regulated significantly. (TIF 469 kb)

Additional file 3: DE lncRNAs with significant difference compared 4-cell with 2-cell of in vivo embryos. (XLSX 24 kb)

Additional file 4: DE lncRNAs with significant difference compared 4-cell with 2-cell of NTM group. (XLSX 18 kb)

Additional file 5: DE lncRNAs with significant difference compared 2-cell with 1-cell of NTC group. (XLSX 17 kb)

Additional file 6: Heat map show the DE lncRNAs levels in in vivo, NTC and NTM groups. Red represents up-regulated significantly, while green represents down-regulated significantly. (TIF 841 kb)

Additional file 7: DE lncRNAs with significant difference compared 4-cell of NTC with 4-cell of in vivo embryos. (XLSX 14 kb)

Additional file 8: DE lncRNAs with significant difference compared 4-cell of NTM with 4-cell of in vivo embryos. (XLSX 17 kb)

Additional file 9: Genes in red modules by WGCNA. (XLSX 38 kb)

Additional file 10: Genes in salmon modules by WGCNA. (XLSX 33 kb)

Additional file 11: The bar chart of the significantly enriched GO terms of genes in the red module by WGCNA. Gene enrichment analysis was carried out by using Metascape, a free online tool for gene annotation (http://metascape.org/gp/index.html#/main/step1). “Log10(P)” is the *p*-value in log base 10. “Log10(q)” is the multi-test adjusted *p*-value in log base 10. (TIF 648 kb)

Additional file 12: The bar chart of the significantly enriched GO terms of genes in the salmon module by WGCNA. Gene enrichment analysis was carried out by using Metascape, a free online tool for gene annotation (http://metascape.org/gp/index.html#/main/step1). “Log10(P)” is the *p*-value in log base 10. “Log10(q)” is the multi-test adjusted *p*-value in log base 10. (TIF 681 kb)

Additional file 13: The bar chart of the significantly enriched GO terms of trans target genes of novel lncRNAs. Gene enrichment analysis was carried out by using Metascape, a free online tool for gene annotation (http://metascape.org/gp/index.html#/main/step1). “Log10(P)” is the *p*-value in log base 10. “Log10(q)” is the multi-test adjusted *p*-value in log base 10. (TIF 591 kb)

Additional file 14: Primer sequence used in amplified cDNA of all samples. (DOCX 13 kb)

## Abbreviations

CC: Cumulus cell
Esc: Embryonic stem cell
FPKM: Fragments per kilobase million mapped reads
GO: Gene Ontology
KEGG: Kyoto Encyclopedia of Genes and Genomes
MEF: Mouse embryonic fibroblasts
NTC: CCs donor NT embryo
NTM: MEF donor NT embryo
OET: Oocyte-to-embryo transition
ORF: Open reading frame
OSKM: Oct4, Sox2, Klf4, c-Myc
PCA: Principal component analysis
PED: Pre-implantation embryonic development
SCNT: Somatic cell nuclear transfer
scRNA-seq: Single-cell RNA sequencing
TOM: Topological overlap matrix
WGCNA: Weighted correlation network analysis
ZGA: Zygotic genome activation

## References

[1] Hemberger M, Dean W, Reik W (2009). Epigenetic dynamics of stem cells and cell lineage commitment: digging Waddington's canal. Nat Rev Mol Cell Biol.

[2] Iwafuchi-Doi M, Zaret KS (2014). Pioneer transcription factors in cell reprogramming. Genes Dev.

[3] Kishigami S, Wakayama T (2009). Somatic cell nuclear transfer in the mouse. Methods Mol Biol.

[4] Wilmut I, Schnieke AE, McWhir J, Kind AJ, Campbell KH (1997). Viable offspring derived from fetal and adult mammalian cells. Nature.

[5] Wilmut I, Bai Y, Taylor J (2015). Somatic cell nuclear transfer: origins, the present position and future opportunities. Philos Trans R Soc Lond Ser B Biol Sci.

[6] Takahashi K, Yamanaka S (2006). Induction of pluripotent stem cells from mouse embryonic and adult fibroblast cultures by defined factors. Cell.

[7] Behringer R, Gertsenstein M, Nagy KV, Nagy A. Reprogramming mouse fibroblasts with *piggyBac* transposons. Cold Spring Harb Protoc. 2017;(10):pdb.prot092627. 10.1101/pdb.prot092627 28974653

[8] Chen J, Chen X, Li M, Liu X, Gao Y, Kou X, Zhao Y, Zheng W, Zhang X, Huo Y, Chen C, Wu Y, Wang H, Jiang C, Gao S (2016). Hierarchical Oct4 binding in concert with primed epigenetic rearrangements during somatic cell reprogramming. Cell Rep.

[9] Wang Y, Bi Y, Gao S (2017). Epigenetic regulation of somatic cell reprogramming. Curr Opin Genet Dev.

[10] Liu X, Wang C, Liu W, Li J, Li C, Kou X, Chen J, Zhao Y, Gao H, Wang H, Zhang Y, Gao Y, Gao S (2016). Distinct features of H3K4me3 and H3K27me3 chromatin domains in pre-implantation embryos. Nature.

[11] Zhuang Q, Li W, Benda C, Huang Z, Ahmed T, Liu P, Guo X, Ibañez DP, Luo Z, Zhang M, Abdul MM, Yang Z, Yang J, Huang Y, Zhang H, Huang D, Zhou J, Zhong X, Zhu X, Fu X, Fan W, Liu Y, Xu Y, Ward C, Khan MJ, Kanwal S, Mirza B, Tortorella MD, Tse HF, Chen J, Qin B, Bao X, Gao S, Hutchins AP, Esteban MA (2018). NCoR/SMRT co-repressors cooperate with c-MYC to create an epigenetic barrier to somatic cell reprogramming. Nat Cell Biol.

[12] Glanzner WG, Rissi VB, de Macedo MP, Mujica LKS, Gutierrez K, Bridi A, de Souza JRM, Gonçalves PBD, Bordignon V (2018). Histone 3 lysine 4, 9 and 27 demethylases expression profile in fertilized and cloned bovine and porcine embryos. Biol Reprod.

[13] Niwa H. The principles that govern transcription factor network functions in stem cells. Development. 2018;145: dev157420. 10.1242/dev.157420 29540464

[14] Herriges MJ, Swarr DT, Morley MP, Rathi KS, Peng T, Stewart KM, Morrisey EE (2014). Long noncoding RNAs are spatially correlated with transcription factors and regulate lung development. Genes Dev.

[15] Peng L, Paulson A, Li H, Piekos S, He X, Li L, Zhong XB (2014). Developmental programming of long non-coding RNAs during postnatal liver maturation in mice. PLoS One.

[16] Grote P, Wittler L, Hendrix D, Koch F, Währisch S, Beisaw A, Macura K, Bläss G, Kellis M, Werber M, Herrmann BG (2013). The tissue-specific lncRNA Fendrr is an essential regulator of heart and body wall development in the mouse. Dev Cell.

[17] Sun J, Lin Y, Wu J (2013). Long non-coding RNA expression profiling of mouse testis during postnatal development. PLoS One.

[18] Bao J, Wu J, Schuster AS, Hennig GW, Yan W (2013). Expression profiling reveals developmentally regulated lncRNA repertoire in the mouse male germline. Biol Reprod.

[19] Qiu JJ, Ren ZR, Yan JB (2016). Identification and functional analysis of long non-coding RNAs in human and mouse early embryos based on single-cell transcriptome data. Oncotarget.

[20] Li Z, Ouyang H, Zheng M, Cai B, Han P, Abdalla BA, Nie Q, Zhang X (2017). Integrated analysis of long non-coding RNAs (LncRNAs) and mRNA expression profiles reveals the potential role of LncRNAs in skeletal muscle development of the chicken. Front Physiol.

[21] Ren H, Wang CL, Jiang J, Liu L, Li N, Zhao J, Sun X, Zhou P (2016). Genome-wide analysis of long non-coding RNAs at early stage of skin pigmentation in goats (Capra hircus). BMC Genomics.

[22] Kopp F, Mendell JT (2018). Functional classification and experimental dissection of long noncoding RNAs. Cell.

[23] Petropoulos S, Edsgärd D, Reinius B, Deng Q, Panula SP, Codeluppi S, Plaza Reyes A, Linnarsson S, Sandberg R, Lanner F (2016). Single-cell RNA-Seq reveals lineage and X chromosome dynamics in human preimplantation embryos. Cell.

[24] Yin Y, Yan P, Lu J, Song G, Zhu Y, Li Z, Zhao Y, Shen B, Huang X, Zhu H, Orkin SH, Shen X (2015). Opposing roles for the lncRNA haunt and its genomic locus in regulating HOXA gene activation during embryonic stem cell differentiation. Cell Stem Cell.

[25] Matoba S, Liu Y, Lu F, Iwabuchi KA, Shen L, Inoue A, Zhang Y (2014). Embryonic development following somatic cell nuclear transfer impeded by persisting histone methylation. Cell.

[26] Liu Z, Cai Y, Wang Y, Nie Y, Zhang C, Xu Y, Zhang X, Lu Y, Wang Z, Poo M, Sun Q (2018). Cloning of macaque monkeys by somatic cell nuclear transfer. Cell.

[27] Fan J, Xing Y, Wen X, Jia R, Ni H, He J, Ding X, Pan H, Qian G, Ge S, Hoffman AR, Zhang H, Fan X (2015). Long non-coding RNA ROR decoys gene-specific histone methylation to promote tumorigenesis. Genome Biol.

[28] Flynn RA, Chang HY (2014). Long noncoding RNAs in cell-fate programming and reprogramming. Cell Stem Cell.

[29] Han P, Chang CP (2015). Long non-coding RNA and chromatin remodeling. RNA Biol.

[30] Merry CR, Forrest ME, Sabers JN, Beard L, Gao XH, Hatzoglou M, Jackson MW, Wang Z, Markowitz SD, Khalil AM (2015). DNMT1-associated long non-coding RNAs regulate global gene expression and DNA methylation in colon cancer. Hum Mol Genet.

[31] Karlic R, Ganesh S, Franke V, Svobodova E, Urbanova J, Suzuki Y, Aoki F, Vlahovicek K, Svoboda P (2017). Long non-coding RNA exchange during the oocyte-to-embryo transition in mice. DNA Res.

[32] Hamazaki N, Uesaka M, Nakashima K, Agata K, Imamura T (2015). Gene activation-associated long noncoding RNAs function in mouse preimplantation development. Development.

[33] Guo F, Li L, Li J, Wu X, Hu B, Zhu P, Wen L, Tang F (2017). Single-cell multi-omics sequencing of mouse early embryos and embryonic stem cells. Cell Res.

[34] Wu J, Xu J, Liu B, Yao G, Wang P, Lin Z, Huang B, Wang X, Li T, Shi S, Zhang N, Duan F, Ming J, Zhang X, Niu W, Song W, Jin H, Guo Y, Dai S, Hu L, Fang L, Wang Q, Li Y, Li W, Na J, Xie W, Sun Y (2018). Chromatin analysis in human early development reveals epigenetic transition during ZGA. Nature.

[35] Li L, Guo F, Gao Y, Ren Y, Yuan P, Yan L, Li R, Lian Y, Li J, Hu B, Gao J, Wen L, Tang F, Qiao J (2018). Single-cell multi-omics sequencing of human early embryos. Nat Cell Biol.

[36] Xu Y, Lian Y, Zhang Y Huang S, Zuo Q, Yang N, Chen Y, Wu D, Sun L.The long non-coding RNA PVT1 represses ANGPTL4 transcription through binding with EZH2 in trophoblast cell. J Cell Mol Med. 2018; 22:1272–1282. 10.1111/jcmm.13405 PMC5783862 29193797

[37] Zhao Q, Guo Z, Piao S, Wang C, An T (2015). Discovery of porcine maternal factors related to nuclear reprogramming and early embryo development by proteomic analysis. Proteome Sci.

[38] Kang E, Wu G, Ma H, Li Y, Tippner-Hedges R, Tachibana M, Sparman M, Wolf DP, Schöler HR, Mitalipov S (2014). Nuclear reprogramming by interphase cytoplasm of two-cell mouse embryos. Nature.

[39] Xue Z, Huang K, Cai C, Cai L, Jiang CY, Feng Y, Liu Z, Zeng Q, Cheng L, Sun YE, Liu JY, Horvath S, Fan G (2013). Genetic programs in human and mouse early embryos revealed by single-cell RNA sequencing. Nature.

[40] Zhang K, Huang K, Luo Y, Li S (2014). Identification and functional analysis of long non-coding RNAs in mouse cleavage stage embryonic development based on single cell transcriptome data. BMC Genomics.

[41] Wang J, Li X, Wang L, Li J, Zhao Y, Bou G, Li Y, Jiao G, Shen X, Wei R, Liu S, Xie B, Lei L, Li W, Zhou Q, Liu Z (2016). A novel long intergenic noncoding RNA indispensable for the cleavage of mouse two-cell embryos. EMBO Rep.

[42] Gao Y, Liu X, Tang B, Li C, Kou Z, Li L, Liu W, Wu Y, Kou X, Li J, Zhao Y, Yin J, Wang H, Chen S, Liao L, Gao S (2017). Protein expression landscape of mouse embryos during pre-implantation development. Cell Rep.

[43] Tang Q, Zheng X, Zhang J (2018). Long non-coding RNA CRNDE promotes heptaocellular carcinoma cell proliferation by regulating PI3K/Akt /β-catenin signaling. Biomed Pharmacother.

[44] Li Z, Tang Y, Xing W, Dong W, Wang Z (2018). LncRNA, CRNDE promotes osteosarcoma cell proliferation, invasion and migration by regulating Notch1 signaling and epithelial-mesenchymal transition. Exp Mol Pathol.

[45] Lv J, Liu H, Yu S, Liu H, Cui W, Gao Y, Zheng T, Qin G, Guo J, Zeng T, Han Z, Zhang Y, Wu Q (2015). Identification of 4438 novel lincRNAs involved in mouse pre-implantation embryonic development. Mol Gen Genomics.

[46] Zhang B, Zheng H, Huang B, Li W, Xiang Y, Peng X, Ming J, Wu X, Zhang Y, Xu Q, Liu W, Kou X, Zhao Y, He W, Li C, Chen B, Li Y, Wang Q, Ma J, Yin Q, Kee K, Meng A, Gao S, Xu F, Na J, Xie W (2016). Allelic reprogramming of the histone modification H3K4me3 in early mammalian development. Nature.

[47] Wu FR, Liu Y, Shang MB, Yang XX, Ding B, Gao JG, Wang R, Li WY (2012). Differences in H3K4 trimethylation in in vivo and in vitro fertilization mouse preimplantation embryos. Genet Mol Res.

[48] McEwen KR, Leitch HG, Amouroux R, Hajkova P (2013). The impact of culture on epigenetic properties of pluripotent stem cells and pre-implantation embryos. Biochem Soc Trans.

[49] Chen YH, Yu J (2015). Epigenetic disruptions of histone signatures for the trophectoderm and inner cell mass in mouse parthenogenetic embryos. Stem Cells Dev.

[50] Bertoldo MJ, Locatelli Y, O'Neill C, Mermillod P (2015). Impacts of and interactions between environmental stress and epigenetic programming during early embryo development. Reprod Fertil Dev.

[51] Liu Y, Wu F, Zhang L, Wu X, Li D, Xin J, Xie J, Kong F, Wang W, Wu Q, Zhang Di, Wang R, Li W. Transcriptional defects and reprogramming barriers in somatic cell nuclear reprogramming as revealed by single-cell RNA sequencing. BMC Genomics. Revised (GICS-D-18-00800). 10.1186/s12864-018-5091-1 PMC6180508 30305014

[52] Tang F, Barbacioru C, Nordman E, Li B, Xu N, Bashkirov VI, Lao K, Surani MA (2010). RNA-Seq analysis to capture the transcriptome landscape of a single cell. Nat Protoc.

[53] Trombetta JJ, Gennert D, Lu D, Satija R, Shalek AK, Regev A (2014). Preparation of single-cell RNA-Seq libraries for next generation sequencing. Curr Protoc Mol Biol.

[54] Picelli S, Faridani OR, Björklund AK, Winberg G, Sagasser S, Sandberg R (2014). Full-length RNA-seq from single cells using smart-seq2. Nat Protoc.

[55] Langfelder P, Horvath S (2008). WGCNA: an R package for weighted correlation network analysis. BMC Bioinformatics.

