# Supplementary figures and images for: Long non-coding RNAs potentially function synergistically in the cellular reprogramming of SCNT embryos
Source: BMC Genomics. 2018 Aug 23;19:631. doi: 10.1186/s12864-018-5021-2 (PMC6107955; doi:10.1186/s12864-018-5021-2)

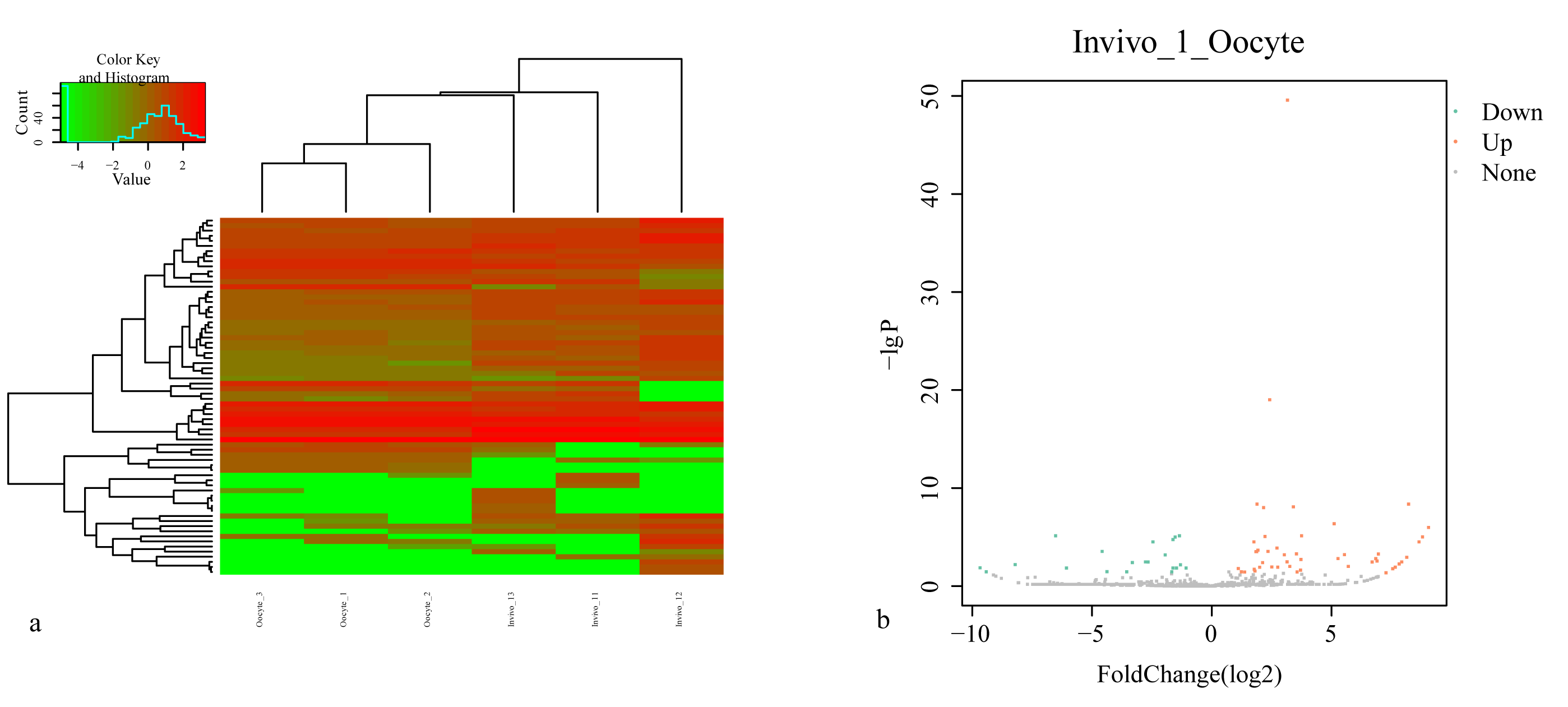

Supplement: Supplementary file 2 — Heat map (a) and Volcano map (b) show the DE lncRNAs levels when compared with oocyte and zygote. Red represents up-regulated significantly, while green represents down-regulated significantly. (TIF 469 kb) [file 12864_2018_5021_MOESM2_ESM.tif]

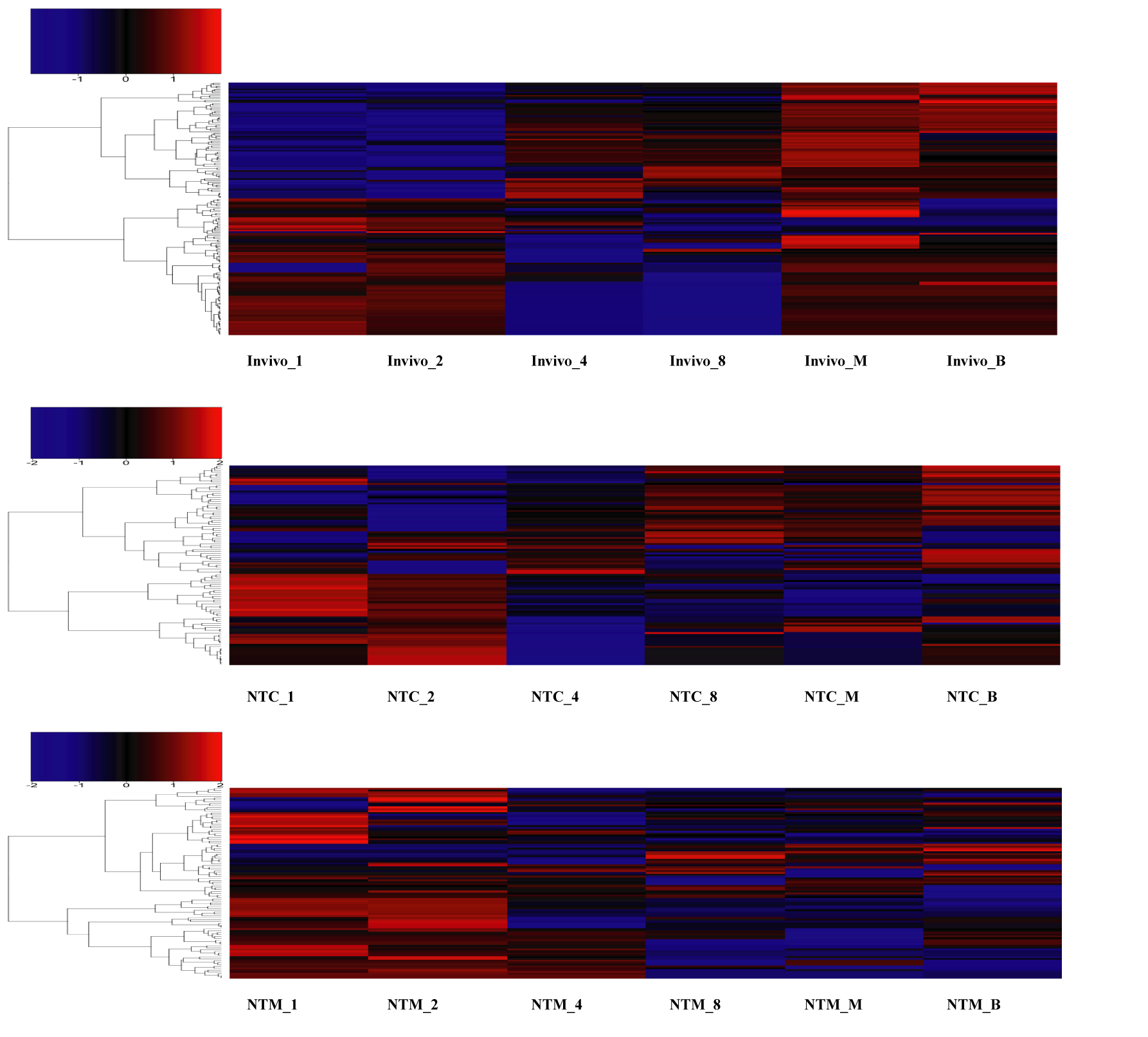

Supplement: Supplementary file 6 — Heat map show the DE lncRNAs levels in in vivo, NTC and NTM groups. Red represents up-regulated significantly, while green represents down-regulated significantly. (TIF 841 kb) [file 12864_2018_5021_MOESM6_ESM.tif]

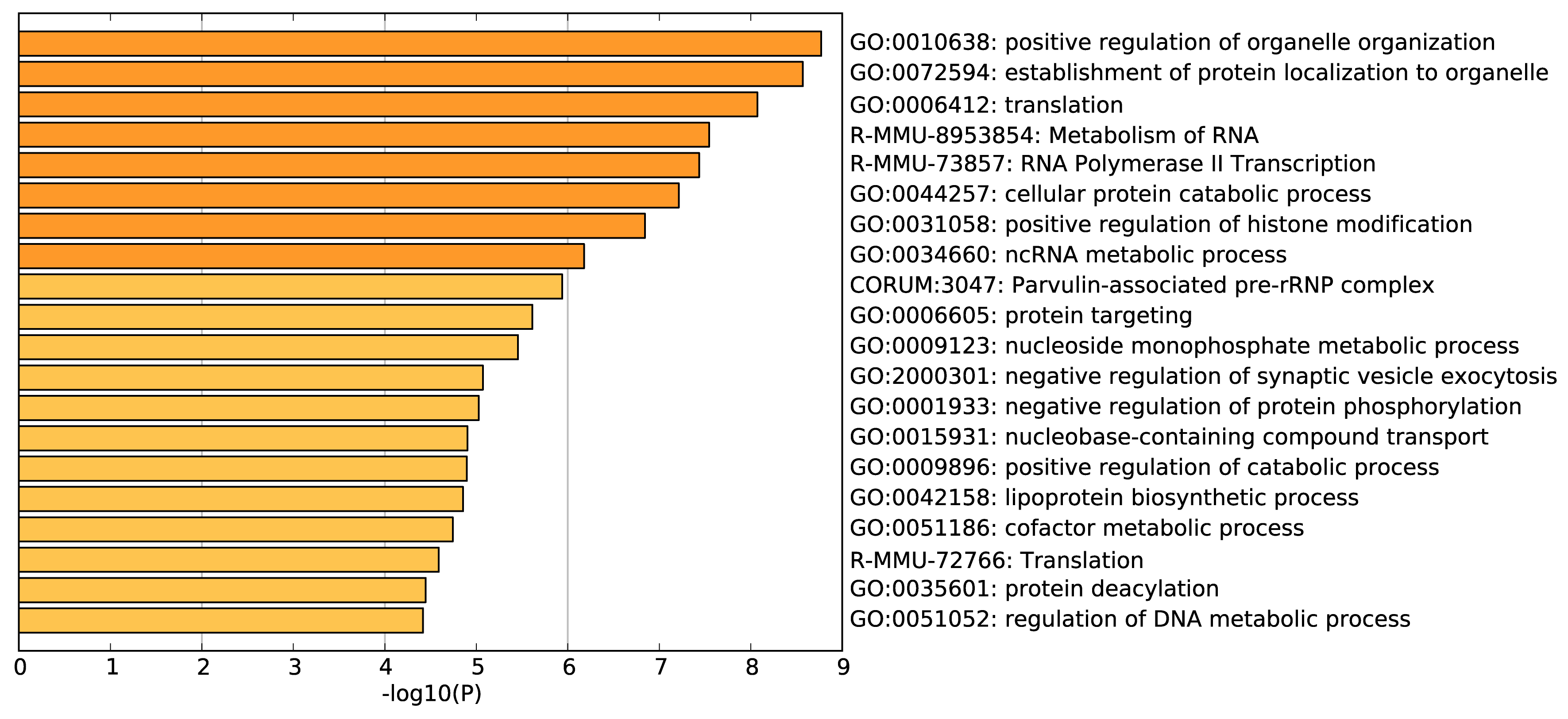

Supplement: Supplementary file 11 — The bar chart of the significantly enriched GO terms of genes in the red module by WGCNA. Gene enrichment analysis was carried out by using Metascape, a free online tool for gene annotation (http://metascape.org/gp/index.html#/main/step1). “Log10(P)” is the p-value in log base 10. “Log10(q)” is the multi-test adjusted p-value in log base 10. (TIF 648 kb) [file 12864_2018_5021_MOESM11_ESM.tif]

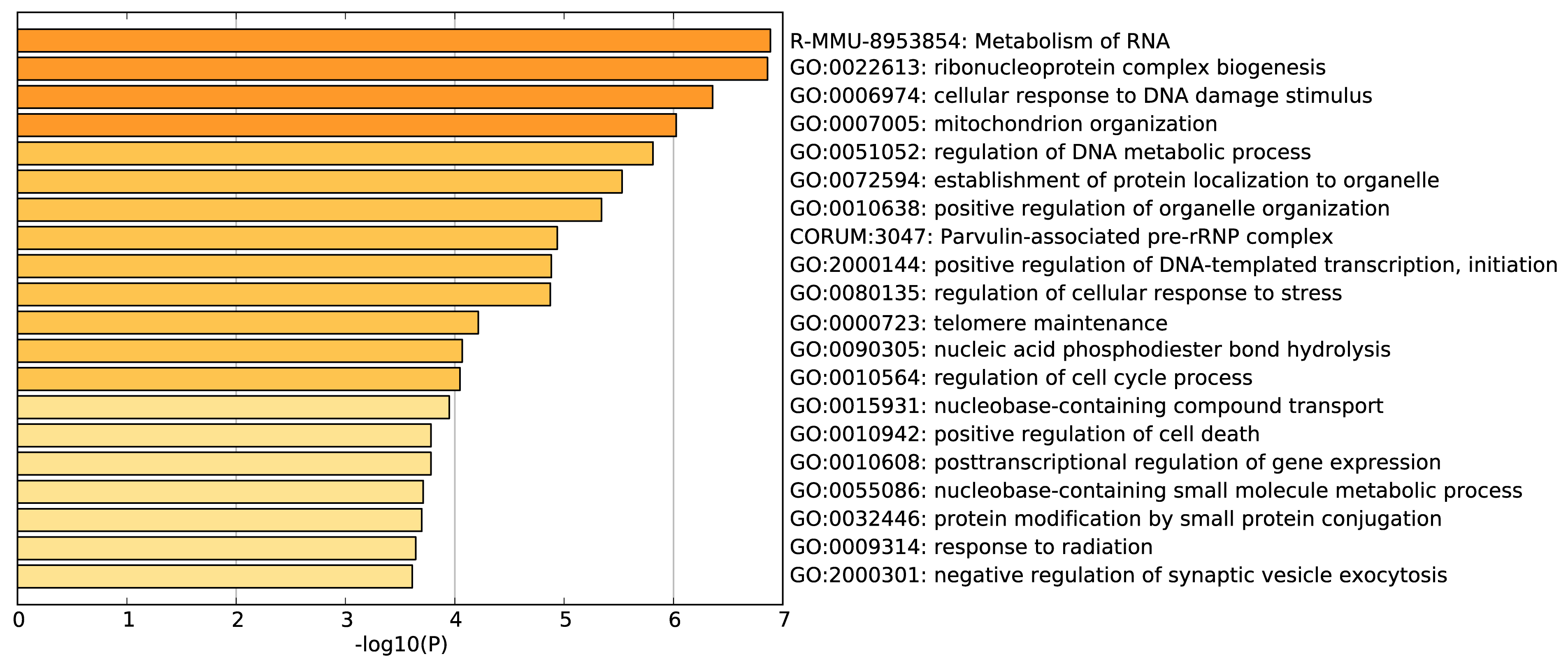

Supplement: Supplementary file 12 — The bar chart of the significantly enriched GO terms of genes in the salmon module by WGCNA. Gene enrichment analysis was carried out by using Metascape, a free online tool for gene annotation (http://metascape.org/gp/index.html#/main/step1). “Log10(P)” is the p-value in log base 10. “Log10(q)” is the multi-test adjusted p-value in log base 10. (TIF 681 kb) [file 12864_2018_5021_MOESM12_ESM.tif]

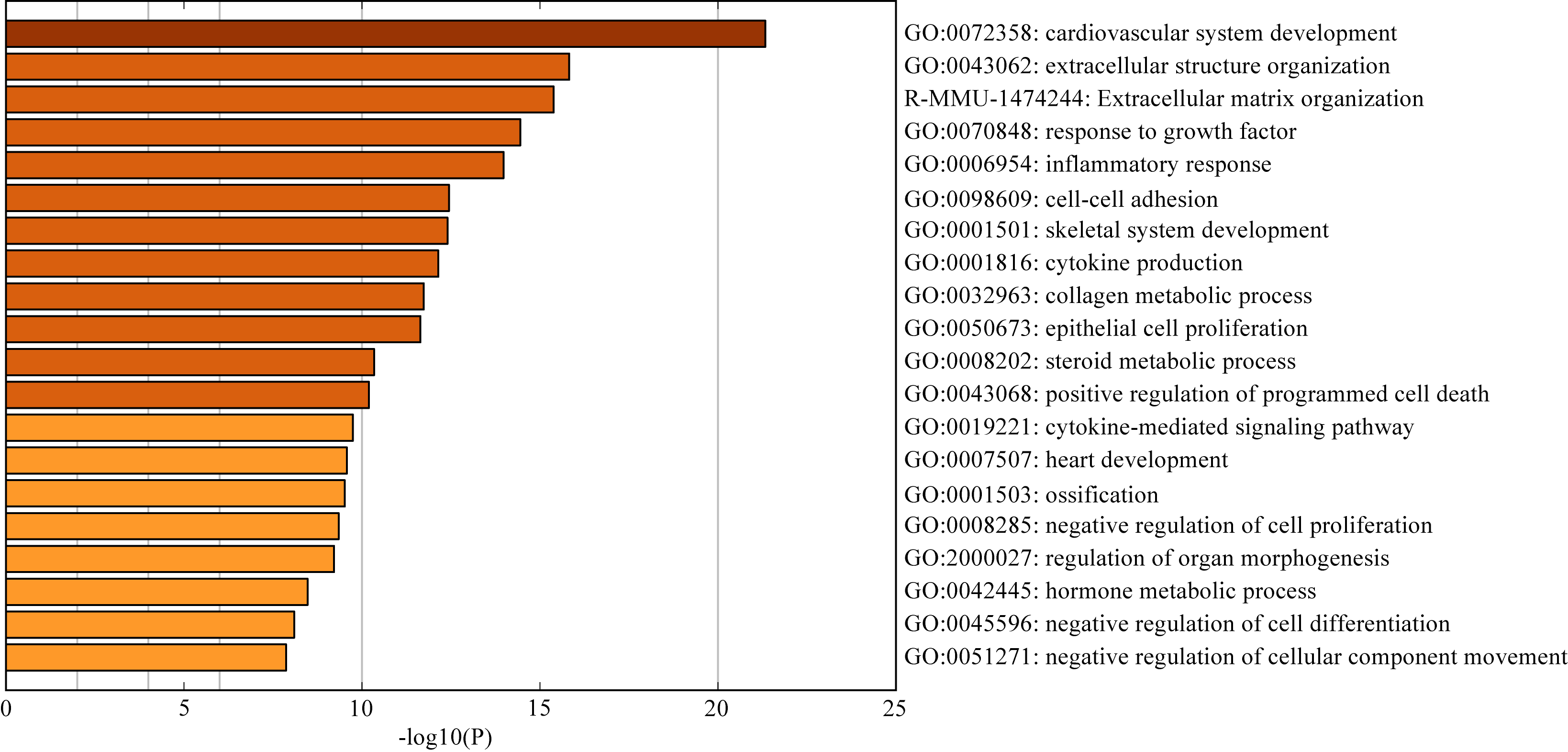

Supplement: Supplementary file 13 — The bar chart of the significantly enriched GO terms of trans target genes of novel lncRNAs. Gene enrichment analysis was carried out by using Metascape, a free online tool for gene annotation (http://metascape.org/gp/index.html#/main/step1). “Log10(P)” is the p-value in log base 10. “Log10(q)” is the multi-test adjusted p-value in log base 10. (TIF 591 kb) [file 12864_2018_5021_MOESM13_ESM.tif]
