# Supplementary material for: Long non-coding RNAs potentially function synergistically in the cellular reprogramming of SCNT embryos
Source: BMC Genomics. 2018 Aug 23;19:631. doi: 10.1186/s12864-018-5021-2 (PMC6107955; doi:10.1186/s12864-018-5021-2)
Supplement: Supplementary file 14 — Primer sequence used in amplified cDNA of all samples. (DOCX 13 kb) [file 12864_2018_5021_MOESM14_ESM.docx]

Table S1 Primer sequence used in amplified cDNA of Single-cell

| Name | Sequence |
| --- | --- |
| UP1 | ATATGGATCCGGCGCGCCGTCGACTTTTTTTTTTTTTTTTTTTTTTTT |
| UP2 | ATATCTCGAGGGCGCGCCGGATCCTTTTTTTTTTTTTTTTTTTTTTTT |
| AUP1 | (NH2)ATATGGATCCGGCGCGCCGTCGACTTTTTTTTTTTTTTTTTTTTTTTT |
| AUP2 | (NH2)ATATCTCGAGGGCGCGCCGGATCCTTTTTTTTTTTTTTTTTTTTTTTT |
